# In Vitro Antifungal Activity of Octenidine Against Emerging Dermatophyte Species

**DOI:** 10.1007/s11046-026-01084-3

**Published:** 2026-06-16

**Authors:** Kathrin Spettel, Richard Kriz, Sarah Fellhuber, Madita Loy, Hannah Tiefenbrunner, Melanie Schabransky, Sonia Galazka, Birgit Willinger

**Affiliations:** 1 Department of Laboratory Medicine, Division of Clinical Microbiology, Medical University of Vienna, Vienna, Austria; 2 Health Sciences, Section Biomedical Science, University of Applied Sciences Campus Wien, Vienna, Austria; 3 Department for Integrative Risk Assessment, Division for Risk Assessment, Data and Statistics, AGES - Austrian Agency for Health and Food Safety, Vienna, Austria

**Keywords:** Dermatophytes, Antiseptics, Octenidine, Terbinafine, Resistance

## Abstract

Dermatophytes cause a wide range of superficial infections of the skin, hair and nails, with high global prevalence and considerable public health relevance. Treatment regimens for dermatophytosis are limited to few antifungal drug classes, and rising resistance—particularly to terbinafine—further compromises therapeutic efficacy. Topical antiseptics such as octenidine (OCT) may represent a promising option for infection control and local therapy. This study investigated the in vitro antifungal activity of the pure antiseptic OCT (at final assay concentrations of 0.1% and 0.05%) and two OCT-based commercial ready-to-use medicinal products (octeniderm® and octenisept®) against clinical isolates of emerging (drug-resistant) dermatophytes including *Trichophyton rubrum, T. mentagrophytes, T. tonsurans, T. interdigitale, T. indotineae* and *Microsporum canis*. Quantitative suspension assays were conducted according to EN 13624:2013 under low organic load (0.3 g/L bovine serum albumin) and high organic load (3 g/L bovine serum albumin, 3 mL/L defibrinated sheep blood), with contact times ranging from 1 to 15 min. Results showed that the active OCT itself as well as the skin antiseptic octeniderm® and the wound and mucous membrane antiseptic octenisept® are effective against the tested dermatophytes in a time- and concentration-dependent manner. Notably, octeniderm® was able to inhibit fungal growth within 1 min, even under high organic load conditions. These in vitro findings suggest that OCT, a well-tolerated antiseptic already in clinical use represents a potential option for preventing transmission and managing superficial infections caused by (drug-resistant) dermatophytes

## Introduction

Dermatophytosis is a highly prevalent infection of global public health concern caused by filamentous fungi belonging to the genera *Trichophyton*, *Microsporum* or *Epidermophyton*, respectively, that can invade keratinized tissues and cause superficial infections of the skin, nails and hair. Clinical manifestations include onychomycosis (nail infection), tinea pedis (athlete’s foot) and inflammatory forms such as tinea corporis, tinea capitis, tinea barbae (skin, scalp and beard ringworm, respectively) or tinea cruris (jock itch). Infections with dermatophytes represent the most common mycosis globally, affecting approximately 20% to 25% of the population [1] and are associated with substantial burden of symptoms including painful inflammatory lesions and unpleasant pruritic rashes. Especially the rising incidence of sexually transmitted dermatophyte infections across multiple countries highlights the urgent need for enhanced clinical vigilance and awareness [2].

A comprehensive understanding of the diversity and pathogenicity of dermatophytes is crucial for effective diagnosis, optimal treatment and infection control [3]. These fungal pathogens are mainly transmitted through direct contact with animals (zoophilic organisms) and/or other people (anthropophilic organisms), as well as indirectly via fomites. In addition, autoinfection from already colonized sites to other body regions may represent a secondary transmission route [4]. Dermatophytosis provoked by different species vary in clinical presentation and thus, pose different therapeutic challenges. Zoophilic fungi such as *Trichophyton mentagrophytes* and *Microsporum canis,* primarily affect the skin and scalp, triggering pronounced inflammatory immune responses, which may be associated with severe symptoms, such as painful erythematous plaques, pustules and nodules. By contrast, anthropophilic species, such as *T. rubrum*, *T. interdigitale* and *T. tonsurans,* typically cause well-demarcated pruritic, scaly skin lesions that are generally associated with limited inflammation. Depending on its severity and extend, dermatophytosis is commonly managed with topical and/or oral antifungal drugs (e.g. allylamines, azoles). However, there has been a concerning increase in recalcitrant chronic and relapsing infections over the past years, which are often associated with unusual clinical presentations and/or mode of transmission [5]. This trend might be further attributed to the rapid emergence of antifungal resistance, which constitutes a growing clinical concern for successful patient outcome [6, 7].

Recently, *T. indotineae* (also named *T. mentagrophytes* genotype VIII) was identified as a novel dermatophyte species with anthropophilic transmission in India, responsible for difficult-to-treat outbreaks currently spreading to many parts of the world [8, 9]. Lesions caused by *T. indotineae* present as extensive inflammatory lesions associated with intense pruritic and a burning sensation. This increasingly prevalent dermatophyte poses significant therapeutic complexity due to its elevated resistance to antifungal agents – particularly terbinafine – driven by target mutations in the squalene epoxidase gene *ERG1*, the fungal orthologue of SQLE [10–12]. Terbinafine has served as a first-line therapy for dermatophytosis since the 1990s, due to its potent antifungal activity and favorable safety profile [12, 13]. However, topical terbinafine, frequently formulated in fixed-dose combinations with corticosteroids, is easily available without prescription in many countries. In that context, unnecessary, unregulated and prolonged use has been linked to the emergence and dissemination of terbinafine-resistant dermatophyte strains [14]. The increasing prevalence of resistance complicates clinical management and underscores the urgent need for novel antifungal agents and alternative therapeutic strategies.

Therefore, antiseptics may represent a viable option for the prevention of transmission and for the localized treatment of recalcitrant dermatophytosis, thereby contributing to limiting the spread of (drug-resistant) dermatophytes. However, actual evidence on the efficacy of clinically used antiseptics against this emerging fungal species remains scarce, mainly due to their slow growth rates and the associated difficulties in performing quantitative suspension tests, which are commonly used for antiseptic efficacy testing, with these strains. Octenidine (OCT), a topically applied antiseptic available in various formulations for diverse medical indications, is recognized for its broad spectrum of antimicrobial activity against a wide range of bacteria [15–20] and yeasts [21–26], including drug-resistant strains. Early clinical reports describe its use as adjunctive therapy for dermatophytosis in a neonate [27] and in a veterinary case involving a rabbit [28]. In addition, a single in vitro study has demonstrated OCT’s activity against one *T. interdigitale* and one *T. rubrum* isolate from onychomycosis patients [29]. Based on these few promising preliminary findings and aiming to expand the current knowledge on the potential of OCT in managing superficial dermatophyte infections, the present study investigated the in vitro efficacy of the pure antiseptic active OCT as well as of two commercial OCT-based pharmaceuticals (octeniderm® and octenisept®) against clinical isolates of emerging (drug-resistant) *Trichophyton* species and *M. canis*.

## Materials and Methods

### Dermatophyte Isolates and Antifungal Susceptibility Testing

A total number of fifteen clinically characterized dermatophyte isolates derived from the culture collection of the Medical University of Vienna, Department of Laboratory Medicine, Division of Clinical Microbiology, Austria, were included in the present study: two *M. canis* (D266, D290), three *T. indotineae* (D299, D301, F1366) two of which harboured terbinafine resistance-associated mutations in *ERG1*: F397L and L393S, respectively, two *T. interdigitale* (D286, D293), one *T. mentagrophytes* genotype VII (D267), one *T. mentagrophytes* genotype III* (D285), four *T. rubrum* (D271, D288, D300, F1396) including one isolate with the F397I mutation in *ERG1* and two *T. tonsurans* (D270, D274) (Table 1). Antifungal susceptibility testing (AFST) was performed according to the European Committee on Antimicrobial Susceptibility Testing (EUCAST) microdilution method for microconidia forming dermatophytes [30] using a panel of eight antifungal agents: amorolfine hydrochloride (0.002–4 mg/L), itraconazole (0.016–32 mg/L), miconazole (0.016–32 mg/L), terbinafine hydrochloride (0.016–32 mg/L), griseofulvin (0.016–32 mg/L), ciclopirox (0.032–64 mg/L), fluconazole (0.125–256 mg/L), and naftifine hydrochloride (0.016–8 mg/L). All isolates were cultured on Sabouraud dextrose agar at 25–28 °C for at least one week prior to testing. Microdilution plates were inoculated with a fungal suspension adjusted to 0.5 McFarland, diluted 1:10 and supplemented with 0.1% Tween-80, 600 mg/L cycloheximide, and 100 mg/L chloramphenicol, in accordance with EUCAST recommendations. Plates were incubated in a humid chamber at 25–28 °C for five days. AFST results were evaluated both spectrophotometrically at 530 nm and visually using a magnifying mirror, with the endpoint defined as a 50% reduction in fungal growth. Due to the frequent occurrence of clump formation, which can compromise spectrophotometric accuracy, visual assessment was prioritized for subsequent determination of minimum inhibitory concentration (MIC) values. Quality control of the microdilution procedure was conducted using the reference strains *Aspergillus flavus* CNM-CM1813, *T. indotineae* SSI-9363/CCUG 74948 and *T. rubrum* SSI-7583/CCUG 74971, following EUCAST guidelines. To date, clinical breakpoints for assessing dermatophyte resistance to specific antifungal agents have not been established as sufficient MIC and clinical outcome data are lacking. However, epidemiological cutoff values (ECOFFs) and proposed thresholds from other studies allow for the interpretation of potential resistance development [31, 32].

**Table 1:** Characteristics of tested dermatophyte isolates including minimum inhibitory concentration (MIC) values in mg/L for depicted antifungal agents

Isolate	Species	*ERG1* Mutation	Genotype	Ecological Niche	AMO	ITR	MCZ	TBF	GRF	CPO	FLC	NFT
D266	*M. canis*	none	NA	ZP	0.064	0.25	0.5	< 0.016	0.5	0.5	32	0.032
D290	*M. canis*	none	NA	ZP	0.032	0.016	0.064	< 0.016	0.125	0.5	8	0.032
D299	*T. indotineae*	none	VIII	AP / ZP	0.125	0.064	0.5	< 0.016	2	0.5	16	0.064
F1366	*T. indotineae*	F397L	VIII	AP / ZP	0.064	0.064	0.125	4	2	0.5	16	> 8
D301	*T. indotineae*	L393S	VIII	AP / ZP	0.064	0.064	0.25	0.5	0.5	0.5	8	0.5
D286	*T. interdigitale*	none	II	AP	0.25	0.064	0.5	< 0.016	0.25	0.5	32	0.016
D293	*T. interdigitale*	none	II	AP	0.125	0.064	0.5	< 0.016	0.5	0.5	32	0.032
D267	*T. mentagrophytes*	none	VII	AP / ZP	0.125	0.125	1	< 0.016	1	0.5	64	0.016
D285	*T. mentagrophytes*	none	III*	ZP	0.125	0.125	1	< 0.016	1	0.5	32	< 0.016
D271	*T. rubrum*	none	NA	AP	0.032	0.064	0.5	< 0.016	2	0.5	32	0.016
D288	*T. rubrum*	none	NA	AP	0.064	0.064	0.125	< 0.016	2	0.5	8	0.016
D300	*T. rubrum*	none	NA	AP	0.25	0.016	0.064	< 0.016	2	0.5	2	0.016
F1396	*T. rubrum*	F393I	NA	AP	0.064	0.064	0.25	0.5	2	0.5	8	1
D270	*T. tonsurans*	none	NA	AP	0.032	0.016	0.5	< 0.016	1	0.5	32	< 0.016
D274	*T. tonsurans*	none	NA	AP	0.032	0.016	0.5	< 0.016	0.5	0.5	32	< 0.016

*ERG1*, squalene epoxidase gene; NA, not applicable; AP, anthropophilic; ZP, zoophilic; AMO, amorolfine hydrochloride; ITR, itraconazole; MCZ, miconazole; TBF, terbinafine hydrochloride; GRF, griseofulvin; CPO, ciclopirox; FLC, fluconazole; NFT, naftifine hydrochloride

### Antiseptic Susceptibility Testing

The antifungal activity of octenidine (OCT) as well as that of the two medicinal products octenisept® (0.1% OCT, 2% phenoxyethanol) and octeniderm® (0.1% OCT, 30% propan-1-ol, 45% propan-2-ol), all obtained from Schülke & Mayr GmbH, Austria, was analysed in quantitative suspension tests according to European Norm (EN) 13624:2013 [16], with an adapted protocol using one-tenth of the standard volumes. This modification enabled high-throughput processing of eight samples simultaneously using an electronic multichannel pipette and 2 mL reaction tubes instead of the conventional 15 mL format. A previous comparison confirmed that the miniaturized set-up yielded results equivalent to those of the standard protocol.

Initially, all dermatophyte isolates were cultured on malt extract agar plates at 30 °C (± 1 °C) for at least one week. Fungal suspensions were prepared by harvesting conidia with a sterile swab into 10 mL of 0.1% Tween-80. After sedimentation of aggregates and centrifugation (5,000 rpm, 10 min), the pellet was resuspended in dilution medium (distilled water, 0.1% tryptone, 0.85% NaCl). In order to remove hyphal fragments and to obtain a predominantly conidial suspension, the sample was filtered sequentially through 100 µm and 11 µm filters. The final fungal concentration (1 × 10⁷ to 5 × 10⁷ cells/mL) was determined by OD₆₀₀ and verified using a LUNA cell counter.

The efficacy of the pure antiseptic OCT at final concentrations of 0.1% and 0.05% as well as of octenisept® and octeniderm® was determined under low organic load (0.3 g/L bovine serum albumin [BSA]) and high organic load (3 g/L BSA and 3 mL/L defibrinated sheep blood), simulating possible organic interference. For each test, 120 µL of the conidial fungal suspension was mixed with 120 µL of respective organic load, followed by 960 µL of the appropriate antiseptic solution. For testing pure OCT at final concentrations of 0.1% and 0.05%, stock solutions of 0.125% and 0.0625% OCT were prepared in distilled water. In contrast, the commercial formulations octenisept® and octeniderm® were used undiluted as ready-to-use products, following the assay protocol specified in EN 13624:2013. After the given contact times ranging from 1 to 15 min, the activity of the antiseptic agent was stopped by adding 960 µL of neutralization solution and 120 µL distilled water. The neutralization solution contained 0.1% tryptone, 0.85% NaCl, 3% Tween 80, 0.3% lecithin, 3% saponin, and 0.1% histidine, and was validated for effective neutralization of the antiseptic without interfering with dermatophyte growth. Subsequently, serial dilutions of the final mixtures were plated on malt extract agar and incubated at 30 °C for 4–7 days. Colonies were counted and the reduction factor (log_10_) was determined as the difference between the number of cells in the test solution at the beginning of the contact time and the amount of recovered colonies after neutralization of the antiseptic activity. A reduction of at least 4 log_10_ was considered indicative of effective antifungal activity according to EN 13624:2013.

## Results

Table 2 summarizes the antifungal efficacy of OCT and the ready-to-use OCT-based formulations octenisept® and octeniderm® against clinical dermatophyte isolates under different organic load conditions and varying contact times. Notably, the alcohol-based skin antiseptic octeniderm® achieved the required ≥ 4 log_10_ reduction for all 15 strains within just 1 min even under high organic load. Otherwise, the aqueous wound and membrane antiseptic octenisept® as well as OCT at concentrations of 0.1% and 0.05% exhibited a time-dependent antifungal activity against most isolates of *Trichophyton* spp. and *M. canis*. In detail, octenisept® showed full efficacy against the majority of dermatophytes (12 out of 15) within 5 min, and against all tested strains within 10 min, regardless of the organic load. Moreover, at a concentration of 0.05%, OCT achieved effective antifungal activity within 10 min under low organic load and within 15 min under high organic load. Taken as a whole, these findings demonstrate a consistent rapid and robust antifungal activity of OCT, octenisept® and octeniderm® against all tested clinical dermatophyte isolates, irrespective of their original ecological niche, mode of transmission or underlying antifungal susceptibility profile.

**Table 2:** log_10_ reduction factor for OCT, octeniderm® and octenisept® under different organic load conditions and given exposure times according to EN 13624:2013.

Substance	Organic load	Contact time	*M. canis*	*M. canis*	*T. indotineae*	*T. indotineae*	*T. indotineae*	*T. interdigitale*	*T. interdigitale*	*T. mentagrophytes*	*T. mentagrophytes*	*T. rubrum*	*T. rubrum*	*T. rubrum*	*T. rubrum*	*T. tonsurans*	*T. tonsurans*
			D266	D290	D299	F1366	D301	D286	D293	D267	D285	D271	D288	D300	F1396	D270	D274
octeniderm®	low	1 min	> 4	> 4	> 4	> 4	> 4	> 4	> 4	> 4	> 4	> 4	> 4	> 4	> 4	> 4	> 4
		3 min	> 4	> 4	> 4	> 4	> 4	> 4	> 4	> 4	> 4	> 4	> 4	> 4	> 4	> 4	> 4
		5 min	> 4	> 4	> 4	> 4	> 4	> 4	> 4	> 4	> 4	> 4	> 4	> 4	> 4	> 4	> 4
		10 min	> 4	> 4	> 4	> 4	> 4	> 4	> 4	> 4	> 4	> 4	> 4	> 4	> 4	> 4	> 4
		15 min	> 4	> 4	> 4	> 4	> 4	> 4	> 4	> 4	> 4	> 4	> 4	> 4	> 4	> 4	> 4
	high	1 min	> 4	> 4	> 4	> 4	> 4	> 4	> 4	> 4	> 4	> 4	> 4	> 4	> 4	> 4	> 4
		3 min	> 4	> 4	> 4	> 4	> 4	> 4	> 4	> 4	> 4	> 4	> 4	> 4	> 4	> 4	> 4
		5 min	> 4	> 4	> 4	> 4	> 4	> 4	> 4	> 4	> 4	> 4	> 4	> 4	> 4	> 4	> 4
		10 min	> 4	> 4	> 4	> 4	> 4	> 4	> 4	> 4	> 4	> 4	> 4	> 4	> 4	> 4	> 4
		15 min	> 4	> 4	> 4	> 4	> 4	> 4	> 4	> 4	> 4	> 4	> 4	> 4	> 4	> 4	> 4
octenisept®	low	1 min	2.39	> 4	3.85	3.07	< 2.67	2.39	> 4	3.72	< 2.47	2.37	> 4	< 2.69	< 2.56	2.67	< 2.18
		3 min	> 4	> 4	> 4	> 4	3.01	3.41	> 4	> 4	2.85	> 4	> 4	3.48	3.38	3.98	2.28
		5 min	> 4	> 4	> 4	> 4	3.56	> 4	> 4	> 4	3.63	> 4	> 4	> 4	> 4	> 4	2.99
		10 min	> 4	> 4	> 4	> 4	> 4	> 4	> 4	> 4	> 4	> 4	> 4	> 4	> 4	> 4	> 4
		15 min	> 4	> 4	> 4	> 4	> 4	> 4	> 4	> 4	> 4	> 4	> 4	> 4	> 4	> 4	> 4
	high	1 min	2.48	> 4	3.77	2.91	< 2.67	2.44	> 4	> 4	< 2.67	2.45	> 4	< 2.69	< 2.56	2.75	< 2.18
		3 min	> 4	> 4	> 4	> 4	2.95	3.82	> 4	> 4	2.87	> 4	> 4	3.42	3.22	> 4	2.32
		5 min	> 4	> 4	> 4	> 4	3.44	> 4	> 4	> 4	3.52	> 4	> 4	> 4	> 4	> 4	3.25
		10 min	> 4	> 4	> 4	> 4	> 4	> 4	> 4	> 4	> 4	> 4	> 4	> 4	> 4	> 4	> 4
		15 min	> 4	> 4	> 4	> 4	> 4	> 4	> 4	> 4	> 4	> 4	> 4	> 4	> 4	> 4	> 4
0.1% OCT	low	1 min	< 2.28	> 4	2.23	< 2.65	< 2.67	< 2.23	3.06	3.69	< 2.67	< 2.51	2.72	< 2.69	< 2.56	< 2.71	2.09
		3 min	3.2	> 4	> 4	3.81	2.8	2.85	> 4	> 4	3.27	3.32	> 4	3.52	2.57	> 4	2.43
		5 min	> 4	> 4	> 4	> 4	3.95	3.71	> 4	> 4	> 4	> 4	> 4	> 4	3.31	> 4	3.13
		10 min	> 4	> 4	> 4	> 4	> 4	> 4	> 4	> 4	> 4	> 4	> 4	> 4	> 4	> 4	> 4
		15 min	> 4	> 4	> 4	> 4	> 4	> 4	> 4	> 4	> 4	> 4	> 4	> 4	> 4	> 4	> 4
	high	1 min	< 2.28	> 4	2.31	< 2.65	< 2.67	< 2.23	2.76	3.76	< 2.67	2.64	2.58	< 2.69	< 2.56	2.59	< 2.13
		3 min	3.03	> 4	> 4	3.59	2.86	2.7	> 4	> 4	3.25	3.86	3.84	3.43	2.76	> 4	2.37
		5 min	3.71	> 4	> 4	> 4	3.74	3.75	> 4	> 4	> 4	> 4	> 4	> 4	3.57	> 4	3.13
		10 min	> 4	> 4	> 4	> 4	> 4	> 4	> 4	> 4	> 4	> 4	> 4	> 4	> 4	> 4	> 4
		15 min	> 4	> 4	> 4	> 4	> 4	> 4	> 4	> 4	> 4	> 4	> 4	> 4	> 4	> 4	> 4
0.05% OCT	low	1 min	< 2.28	> 4	< 2.5	< 2.65	< 2.67	< 2.23	2.78	3.18	< 2.67	< 2.44	2.33	< 2.69	< 2.56	< 2.71	< 2.18
		3 min	2.59	> 4	> 4	3.52	2.69	2.7	> 4	> 4	3.06	2.85	3.44	2.79	2.35	3.84	2.34
		5 min	3.4	> 4	> 4	> 4	3.45	3.53	> 4	> 4	> 4	> 4	> 4	3.73	2.81	> 4	2.82
		10 min	> 4	> 4	> 4	> 4	> 4	> 4	> 4	> 4	> 4	> 4	> 4	> 4	> 4	> 4	> 4
		15 min	> 4	> 4	> 4	> 4	> 4	> 4	> 4	> 4	> 4	> 4	> 4	> 4	> 4	> 4	> 4
	high	1 min	< 2.28	> 4	< 2.5	< 2.65	< 2.67	< 2.23	2.38	3.17	< 2.67	< 2.44	2.23	< 2.69	< 2.56	< 2.71	< 2.18
		3 min	2.42	> 4	3.64	3.03	< 2.67	2.42	3.07	> 4	2.87	< 2.44	2.63	2.48	< 2.56	3.86	2.17
		5 min	2.91	> 4	> 4	3.95	2.74	2.91	> 4	> 4	> 4	2.66	3.34	3.2	2.47	> 4	2.35
		10 min	> 4	> 4	> 4	> 4	3.8	> 4	> 4	> 4	> 4	3.88	> 4	> 4	3.62	> 4	3.33
		15 min	> 4	> 4	> 4	> 4	> 4	> 4	> 4	> 4	> 4	> 4	> 4	> 4	> 4	> 4	> 4

OCT, octenidine; low organic load: 0.3 g/L bovine serum albumin; high organic load: 3 g/L bovine serum albumin + 3 ml/L defibrinated sheep blood; octenisept® and octeniderm® were evaluated at 80% of their recommended concentration for clinical application due to assay setup

## Discussion

The increasing global prevalence of chronic and recurrent superficial infections caused by either drug-susceptible or drug-resistant *Trichophyton* and *Microsporum* species has complicated the management of dermatophytosis, necessitating revised therapeutic strategies for improved patient outcomes. Topical antiseptic agents may serve as cost-effective, easy-to-apply alternatives for localized infection control, even suitable for home use without significant associated health risks. In this regard, the present study investigated the in vitro susceptibility of various dermatophyte isolates against OCT, a clinically well-established antimicrobial molecule used for skin, wound and mucous membrane antisepsis [33–36], treatment of vaginal infections [37–40], as well as microbial decolonization of patients [41–46].

Indeed, quantitative suspension tests performed according to EN 13624:2013 highlight a potent efficacy of the pure antiseptic active OCT as well as of the two commercial OCT-based products octeniderm® and octenisept® against a total number of 15 emerging *T. rubrum, T. mentagrophytes, T. tonsurans, T. interdigitale, T. indotineae* and *M. canis* clinical isolates. OCT retained its antimicrobial activity even under high organic load (3 g/L bovine serum albumin, 3 mL/L defibrinated sheep blood), which is in line with recent in vitro tests performed with drug-resistant bacteria and yeasts based on corresponding European Standards [17–19, 21]. These findings may be particularly relevant for clinical applications, as organic components (e.g. blood, mucin, exudate) present on patient’s skin, wounds and mucous membranes can negatively interfere with the antimicrobial efficacy of other antiseptics (e.g. PVP-iodine, polyhexanide) [47–51]. Furthermore, both OCT-based pharmaceutical formulations were diluted in accordance with the official test norm, which means such ready-to-use products were evaluated at only 80% of the recommended concentration for clinical application. Despite this dilution, both the alcohol-based skin antiseptic octeniderm® and the aqueous wound and mucous membrane antiseptic octenisept® demonstrated full efficacy against all tested dermatophyte isolates within short contact times favouring a practicable clinical use. These findings may support healthcare professionals in choosing appropriate new topical antifungal interventions in the management of dermatophytosis, based on results that meet the standards of EN 13624:2013.

To our knowledge, this is the first comprehensive in vitro study providing evidence that OCT exhibits comparable efficacy across a diverse range of anthropophilic and zoophilic dermatophyte species, involving three highly emerging terbinafine-resistant *Trichophyton* isolates, connected with point mutations F397L and L393S in the *ERG1* gene [10–12]. Importantly, the obtained data further comprises multiple genotypes of arising sexually-transmitted *T. mentagrophytes* such as ITS genotype III*, ITS genotype VII and *T. indotineae,* frequently associated with severe and atypical lesions [2, 52]. OCT’s recently described unspecific mechanism of action based on purely physical interactions with lipid membranes of different bacterial and fungal pathogens [23, 53–56] may represent the basis of its broad antimicrobial profile, and is now also investigated for various clinical relevant (drug-resistant) dermatophyte species. Moreover, it is hypothesized that due to the immediate disruption of highly conserved membrane structures, any development of clinically relevant reduced susceptibility towards OCT or even (cross)resistance to other commonly used antimicrobial agents (antibiotics, antifungals, antiseptics) may be less likely and has not been consistently reported to date [20, 22, 57–62], despite its application in different product formulations for more than 35 years in clinical and outpatient settings. These findings suggest that OCT may have a role as an adjunct or alternative in therapeutic regimens for dermatophyte infections, particularly in cases of underlying antifungal resistance or failure of conventional treatments.

Dermatophytosis often constitutes a diagnostic challenge for healthcare professionals due to an atypical inflammatory appearance (e.g. pustules, papules), which may also resemble a bacterial infection or non-infectious dermatitis [2]. This increases the risk of mismanagement, where patients may empirically undergo one or more unnecessary courses of antibiotics and/or corticosteroids without prior testing for a potential fungal infection, often masking or even worsening preliminary symptoms. Such inappropriate interventions may result in significant morbidity and continuous transmission of the disease. Due to its broad antimicrobial efficacy profile, covering both potential harmful bacterial and fungal species, topical applied OCT may represent a promising option for infection control and local therapy, especially when the diagnosis of the causative pathogen is initially unclear, but also in cases of secondary superficial infections involving various pathogenic microbes.

Apart from OCT’s potent antimicrobial activity, data from clinical trials indicate that different OCT-containing products may positively influence wound healing [35, 36, 63] accompanied with improved scar quality [64]. In this context, recent ex vivo experiments in a human superficial wound model suggest that OCT may exert immunomodulatory effects at the cellular and molecular level, including anti-inflammatory and protease-inhibitory activity [65, 66], which were not observed with certain other antiseptics under comparable conditions [67]. These capabilities may also enable OCT to modulate immune responses within clinical manifestations of dermatophytosis by facilitating the resolution of painful inflammatory lesions and pruritic skin conditions through the downregulation of inflammatory pathways. In addition, compared to other antiseptics, OCT shows a remanent effect of at least 48 h [34], attributed to its lack of systemic absorption through human skin. Importantly, when applied locally, ready-to-use OCT-based medicinal products with a long-established and well-proven history of clinical application are not associated with any severe local and systemic adverse reactions [68], supporting their use in pregnant women and neonates [40, 46]. The two OCT-containing formulations differ in composition and intended use. Octeniderm® is an alcohol-based skin antiseptic for pre-procedural skin disinfection, whereas octenisept® is an aqueous OCT/phenoxyethanol preparation for superficial wound and mucosal antisepsis and time-limited supportive treatment of interdigital mycoses. Therefore, tolerability and suitability should be assessed in relation to the specific indication, application site and duration of use.

There may be some possible limitations in the presented findings that could be addressed to guide future research. Firstly, the in vitro study focused exclusively on a defined set of 15 clinically relevant (drug-resistant) *Trichophyton* spp. and *M. canis* to evaluate their susceptibility to OCT, whereas other dermatophyte species of human health concern such as *Epidermophyton floccosum*, *Nannizia gypsea* or *T. benhamiae* were not included. However, to our knowledge, this represents one of the largest in vitro studies assessing the activity of OCT against dermatophytes. The overall number of tested isolates remains limited, and future studies encompassing a broader panel of dermatophyte species – including *Epidermophyton floccosum*, *Nannizia gypsea*, and *T. benhamiae* – as well as a larger number of clinical isolates per species are warranted to further corroborate these findings.

Secondly, although testing in accordance with EN 13624:2013 provides a standardized and reliable measure of antiseptic efficacy against planktonic dermatophyte forms, it may not fully reflect real-world clinical conditions. Factors such as biofilm formation and hyphal growth, deep dermal invasion or infiltration into keratinized tissues (e.g. nail plates) may substantially enhance fungal resilience and reduce antiseptic effectiveness. To our knowledge, the current literature reports only one human case of tinea capitis in a newborn triggered by *T. soudanense*, where scalp lesions were successfully treated with topical OCT and clotrimazole for one week as an adjunctive therapy [27]. Further comprehensive studies are needed to evaluate the effectiveness of different ready-to use OCT-containing products – particularly those intended for whole-body decolonization or hydrogel formulations in the treatment of superficial dermatophytosis. In this context, potential adverse effects during application in patients with dermatophytosis should always be carefully monitored and assessed by the treating physician within the overall individual benefit–risk evaluation of the respective product in real-life clinical settings.

In summary, the results show that OCT exhibits high antiseptic activity against clinical isolates of emerging (drug-resistant) *Trichophyton* species and *M. canis*. In the spotlight of the increasing global emergence of pathogenic dermatophytes associated with substantial morbidity, the present data support the potential future role of OCT for transmission prevention and an effective therapeutic option for the management of superficial infections, including those caused by drug-resistant strains.

## Data Availability

No datasets were generated or analysed during the current study.
